# Chemical species: the art and politics of living with(out) drugs after addiction

**DOI:** 10.1057/s41292-022-00281-9

**Published:** 2022-09-27

**Authors:** Fay Dennis

**Affiliations:** Goldsmiths, University of London, 10.01 Warmington Tower, 8 Lewisham Way, London, SE14 6NW UK

**Keywords:** Art-based methods, “chemical turn”, Drug use and ‘addiction’, Entanglement, Paying attention

## Abstract

We live within and are made up of ever-changing chemical flows. Witnessing a “chemical turn” in the social sciences, this article asks what a chemical reading of drugs and bodies can offer an understanding of drug dependency and recovery. Where chemicals render bodies “molecular” (Deleuze and Guattari, 1987), they open them up to more intimate forms of connection that extend our understanding of drug–body relationships beyond limiting categories such as addiction. Rather than a chemical drug entering a biological body, there are chemical interactions that expand the boundaries of where one ends and the other begins. While chemicals have long been a preoccupation in neurological models of addiction, they are seldom taken up in sociological studies of these concerns. Drawing on a series of body-mapping workshops with people in drug recovery/treatment in London, UK, to track these chemical bodies, this article explores the art of living a chemically transformed life. This is an art that thinks with Isabelle Stengers’ (in Stengers and Savransky, 2018) notion of the word to include “not paying attention” as a mode of “paying attention to what may lurk” in living with the ongoing effects of drugs in unequally entangled worlds.

## Introduction

In our late industrial epoch, we are embroiled in increasingly complicated networks of chemicals in the air we breathe, water we drink, food we eat, and homes we live in. This has led to what some have called a “chemical turn” in the social sciences, where biological species are being reimagined as chemical species, embedded in these chemical ecologies (e.g. Agard-Jones 2013; 2016; Balayannis and Garnett 2020; Hardon and The Chemical Youth Collective 2017; Lee 2020; Liboiron et al. 2018; Murphy 2008; Povinelli 2016; Roberts 2011; Romero et al. 2017; Shapiro 2015; Shapiro and Kirksey 2017). As this turn radically rethinks our biological form, with chemicals breaking down assumed boundaries between bodies and their environments, there has been surprisingly little attention paid to what chemicals and chemical ecologies can do for rethinking drug consumption practices, dependency, and recovery as overt kinds of chemical practices. While addiction sciences study chemical imbalances in the brain, chemicals are seldom taken seriously for extending and destabilizing these forms as they relate to the social.

Chemicals are known for passing through, collapsing, and otherwise disturbing boundaries. They oscillate, decompose, infiltrate, evaporate, and explode. As the research participant explains (in the epigraph), he is moved and defined by a unique set of chemical reactions pulling him back into the drug use he is trying to escape. Reducing his drug use to the function of chemical reactions, he concludes: “there’s no sense to it”. Rather than assigning agency to himself, he attributes it to a chemical manipulation of the brain. External (‘drug’) and internal (‘brain’) chemicals are entangled, where we find out below, living without drugs is always a way of learning to live *with* them. For this participant, heroin and crack cocaine were uniquely effective at delivering pleasure and relief (“you couldn’t get it any other way”), described as a sensation and memory that resides in the body (“in there”), beneath or beyond “sense”. Recognising this participant’s train of thought, as I believe it was intended, as a provocation (“…or whatever it is going on in there…”), the project I draw on in this article seeks to get closer to and ask questions of these chemical bodies and their perceived senselessness beyond a dominant understanding of addiction as a biomedical problem.

Bodies and drugs as chemicals can be seen to be conjoined in a way that confuses our individualized notion of desire (“my body wants to be rewarded”). Addiction as a “molar” structure, simplifies and shuts down desire, where chemicals may form a more fluid or “molecular” mode of knowing and intervening in these relationships. In making this distinction between molar and molecular structures or articulations, Deleuze and Guattari (1987) state that in the former “an overcoding [is] produced, phenomena of centring, unification, totalization, integration, hierarchization, and finalization” (1987, p. 40). I turn to chemicals as a way of undoing these forms by following the molecular:

If molar unities […] attempt to form and stabilize an identity, a fixity, […] molecular becomings traverse, create a path, destabilise, enable energy seepage within and through these molar unities (Grosz 1998, p. 176, cited in Roy 2018, p. 21).

Like with Deboleena Roy’s practice of “molecular feminism”, which traces the minoritarian work of bacteria as well as other organic and inorganic matter in the laboratory, it is in following these drugged entanglements that I hope to reveal alternative modes of living with(out) drugs to addiction/recovery. With this, I speak to recent calls from prominent critical addiction scholars to “*compose* new directions for thinking and speaking about drug use” (Fraser 2017, p. 131, original emphasis) and “expand our repertoire of creative responses” (Keane 2021, p. 4). This is an attempt to find the cracks and openings in the limiting ‘problem’ of addiction.

As articulated in this journal over a decade ago, addiction operates as a socially and historically specific mode of knowing, ‘molarising’ certain behaviours, consumptions, and desires that are considered excessive and uncontrolled within a medico-ethical framework (Keane and Hamill 2010, p. 53; Dunbar et al. 2010). As a result, addiction over-codes and precludes our thinking on how drugs connect with bodies, with most recent iterations locating this within the brain as a neurological defect (Dunbar et al. 2010; Fraser 2017; Vrecko 2010). As the participant in the epigraph highlights, it becomes difficult to think these connections otherwise, and, like with all identity politics, it becomes hard for people to account for their experiences outside of these identities (Malins 2017; Fraser et al 2017), resulting in ‘self-stigmatizing’ stories (Fraser et al. 2017) and what Peta Malins (2017) calls “fascist” treatment systems where becoming other (than an addict) is “blocked” (see also, Dennis 2019).

Expanding on a methodology of body mapping for storying drugs and the people who use them differently (Dennis 2020), this project seeks to further open-up this ‘black box’ of addiction that rigidifies how we can think and treat drugs, in particular, for those trying to live without them. It explores the complicated practices involved in giving up drugs and learning to live with their ongoing effects as collective, embodied, and kinetic practices (detailed below). With this, the project builds on a growing area of critical drug scholarship that decentres the drug-using subject in recognising the assemblic nature of drug consumption (e.g. Dennis and Farrugia 2017; Duff 2015; Dilkes-Frayne and Duff 2017; Gomart and Hennion 1999; Schipstal et al. 2016; Race 2014) and recovery (e.g. Theodoropoulou 2021; Duff et al. forthcoming). Rather than a biological *problem* becoming known differently over time and space in a “biocultural” explanation of addiction (Dunbar et al. 2010), these scholars document an intra-action of time, space, and bodies where subjectivities and substances co-configure (Fraser and valentine 2006; Fraser et al. 2017; Race 2018; see also Dennis et al. 2020).

Therefore, in an extension of this scholarship and the arguments made in this journal “to overcome the biology/cultural dualism” of addiction (Dunbar et al. 2010, p. 2), I turn to chemical relations and us as chemical species as a way of accounting for bodies, drugs, and societies as already entangled, enabling us to fully account for people’s conflicts and confusions towards their drug use as more than addiction. That is, more than ‘addiction’, both as a biological/neurological affliction *and* as a set of discourses determined by these life sciences (as well as the ‘psy disciplines’ and recovery groups like Alcoholics Anonymous). But more than this, such a reading can also attune us to the unequal nature of these entanglements. That is to say, while psychoactive drugs and a desire to change one’s mental and bodily state have always been with us (e.g. Bancroft 2009; Cultures of Energy 2016; Herring et al. 2013), the forms of violence they intersect with such as prohibition, colonialism, racism, sexism, and poverty mean they have grossly divergent effects. This is witnessed most strikingly in the opioid overdose crisis globally and particularly in North America and the United Kingdom where deaths have followed clear lines of ingrained hostility (e.g. Campbell 2020; Hamilton 2020; Hamilton and Eastwood 2020; Koram 2019).

By turning to chemicals, rather than bodies and drugs as distinct entities, participants in this study depict chemical, ‘drugged’ ecologies as they converge on the body, where they understand themselves as always in a process of recovery but never fully recovered—as a “work in progress”. Rather than a transformation from one clearly defined state to another (such stories of rebirth and arrival are common in drug rehabilitation), there is constant “work”, where these chemical relations are never fully escaped but rather mediated and lived *with*. Through a “cat-cradling” of bodies and things in the workshops, participants describe *artful* ways of paying attention to these chemical attunements characterised as “the addict” that lurks. I borrow, here, from Isabelle Stengers understanding of paying attention as an art—“I call it art […] a rather Quaker art, to ‘bethink’, to pay attention to what may lurk” (Savransky and Stengers 2018, p. 136). But more than a recognition of these enduring and artful modes of living a chemically transformed life (as a possibly more ethical way of thinking about these connections after addiction), this article calls for an understanding of the political implications of such a life “in progress” that requires vigilance—a vigilance that, as we will see, has an unequal history and political economy. To make this argument, let me first turn to the chemical thinking that has made these sensibilities possible.

## Background: chemical species and ecologies

Thinking of chemical species as molecular species (Murphy 2008, c.f. Rose 2007; Roy 2018), we can explore the impact of the environment on the body as already entangled (Barad 2007; Landecker 2016). “At the material metabolic and chemical level, embodiment is already altered, already non-innocently embroiled in uneven violent relations of being and doing” (Murphy 2016: no page). This is vital for understanding the depths that drugs affect us even unknowingly. Chemicals attune us to the porosity of “bodies in the system” as a pressure pot of these connections, where political economies and histories get tied to flesh (Agard-Jones 2013). This is something that gets neglected in addiction discourses that focus on individual pathology, autonomy, and recovery. For mapping chemical infrastructures and bodies, I have been particularly inspired by feminist ethnographers working with and at the molecular such as Deboleena Roy, Michelle Murphy, Vanessa Agard-Jones, Hannah Landecker, Elizabeth Roberts, Nerea Cavillo and Emma Garnett (and to be discussed later in the article, Isabelle Stengers, Maria Puig de la Bellacasa and Eva Giraud). For the purposes of rethinking drug dependency and recovery after addiction, such works attune us to drugs and drugged bodies as they are 1) embedded in chemical ecologies, 2) porous and volatile and 3) in need of new ways of knowing.

Documenting the violent *chemical ecologies* of the Athabasca Chipewyan First Nation who live downstream from the Tar Sands oil fields in western Canada, Michelle Murphy describes how

cars, militarization, water, laws, the direction of a river, the price of oil, the properties of sand, the rise of neoliberalism, histories of colonial dispossession are all part of a complex of *molecular relations* that extend outward in place, and into the past, as well as forward to uncertain futures. (2008, p. 696, original emphasis)

Entitled “chemical regimes of living”, Murphy (2008) explores the profoundly polluting work of extracting oil, linked to a multitude of illnesses including rare cancers. Building on Nikolas Rose’s (2007) molecularization of biopower, Murphy carefully ties political economies to technoscience in which “chemical regimes of living” are not simply the result of new epistemologies but a result of “some two hundred years of industrialized production” (2008, p. 697).

Not only are we experiencing new forms of chemical embodiment that molecularly tie us to local and transnational economies, but so too processed food, hormonally altered meat, and pesticide-dependent crops become the material sustenance of humanity's molecular recomposition. We are further altered by the pharmaceuticals imbibed at record-profit rates, which are then excreted half metabolized back into the sewer to flow back to local bodies of water, and then again redispersed to the populace en masse through the tap. (2008, p. 696)

While these pharmaceutical infrastructures are now well documented (e.g. Banerjee 2016; Dertadian 2018; Dumit 2012; Ecks 2013; Herzberg 2009; 2020; Martin 2006; Sunder 2017), less attention has been paid to illicit drugs in these chemical regimes of living.

Illicit drugs cannot be divorced from the political economies and histories that have driven wars and made some countries and peoples prosper, while others are subjugated by imperialist power, heavily policed, and punished. In the United States, for example, the notorious Anti-Drug Abuse Act of 1986 set out mandatory minimum sentences for crack cocaine as differentiated from powder cocaine which led to vastly longer prison terms for Black people over white people. Living within this “contaminated diversity”, to use Anna Tsing’s (2012) term, as employed by Rhodes et al. (2021) in relation to coca eradication in Columbia, an ecological approach to drug use tries to take account of drug environments and policies as they become-with humans. As Vanessa Agard-Jones (2013, 2014) reminds us in her ethnographic work on pesticide use in Martinique: “at every scale in our social and biological worlds, contingent forms of non-life and life are being entwined, as synthetic chemicals embed, accrete, and leave their residue in our bodies” (2014, no page). As we will see, this kind of chemical thinking is vital for reimagining our public health responses to drugs that have focussed too much on the end user as autonomous.

Moving between these scales of legal/economic/public health policy and illegal substances in her understanding of chemical ecologies in Mexico City, Elizabeth Roberts (2011) tracks chemical exposure and penetration. Robert explores “what gets inside”—including policies such as the War on Drugs and North American Free Trade Agreement and the chemical substances they affect, such as soda, marijuana and *activo*—at both the level of the body and the district. Tying these two scales together, she “examines both the pleasures and the perils of permeability, porosity, and penetration” (2011, p. 597). Taking a critical approach to entanglement, this is not simply an account of complex drug-body-worlds, but a call to take seriously attempts to make boundaries where “for many of the world’s inhabitants, the entanglement of everything with everything proves relentlessly exhausting” (2011, p. 615). This is an extremely pertinent point and one that I return to.

Becoming-with the molecular constituents of various chemicals (unknowingly or involuntarily) speaks to the *porosity of the chemical body* as articulated by participants in this study in rethinking the addicted body. To develop this argument further, I turn to Hannah Landecker (see also Murphy 2016; Povinelli 2016; Shapiro 2015). Tracing our particularly conflicted relationship to one kind of drug, antibiotics, and the growth of antibiotic resistance, Landecker describes a looping effect in which we are “feeding animals and plants to microbes [in the fermenting process of antibiotics] in order to feed antibiotics to animals and plants in order to feed humans who are themselves (relatively speaking) doused in antibiotics” (2016, p. 36). This is an incredibly leaky system in which antibiotics intended to treat one strand of bacteria or even one individual body seep out, mixing not only with other bacterium but other bodies. Landecker writes:

Antibiotics have been called a “societal drug”: when one person in a household takes an antibiotic for an extended period, for example to treat acne, density of antibiotic resistant bacteria on the skin of everyone else in the household increases (Levy 1998). (2016, p. 34)

Where, after Foucault (2008), we have tended to focus on the politics of biology, Landecker incisively argues for a “biology of history” that fully considers the interconnectedness of biology, technology, and culture—what she calls a “biology of twentieth century biopolitics” (2016, p. 43). This translates, here, to a concern with the molecular materiality of bodies, drugs, and technologies. It is by reading drug practices as molecular practices that we can start to unravel addiction as a particular kind of problem operating at the level of the biological body. Therefore, to re-pose Landecker’s observation as a question: *in what ways might “this biological matter, [chew] away its own ontology”?* Or, for our purposes, *in what ways might chemicals erode how we think about addiction?*

To allow such possibilities, we need new ways of attuning to chemicals. Exploring the ways formaldehyde (from common domestic adhesives) gets absorbed into the body, disrupting cellular function, Nicholas Shapiro shows how “indistinct and distributed harms are sublimated into an embodied apprehension of human vulnerability to and entanglements with ordinary toxicity, provoking reflection, disquiet, and contestation” (2015, p. 369). As such, he explores “bodily reasoning” as a way to “know and reimage our ongoing late industrial present” (Shapiro 2015, p. 370). This, I believe, is what the participant quoted in the epigraph is pointing towards in the “senselessness” of his drugged desires, which can become sense-full or rather “sense/ible” (Calvillo and Garnett 2019) if we listen to bodies as ecologically attuned, entangled phenomenon, rather than afflicted matters-of-fact. It is in giving a voice to this bodily sense that this article seeks to make an ethical intervention in how we understand drugs (illicit/licit) and the people who use them.

How chemicals are rendered knowable are “effects of a complex political economy” (Murphy 2008), and thus, finding ways to register them becomes political and ethical work (Barad 2007). For example, in Lancaster et al.’s (2019) interrogation of wastewater analysis as “a more accurate” way of monitoring local drug-use rates, they observe how such technoscience not only alters ways of knowing but in doing so reinforces dominant images of people who use drugs as “both ‘unknowing’ and ‘unworthy knowers’” who cannot be trusted to self-disclose their drug use (2019, p. 51). This corroborates Shapiro and Kirksey’s observation that monitoring systems can “limit conversations to the priorities of scientists, policy makers and corporations” (2017, p. 483). Therefore, going back to the potentially liberating work of bodies, they might be able to disrupt these knowledges.

Following Calvillo and Garnett (2019), bodies are not only sites *for* knowledge but can *open-up* subjugated ways of knowing and being. Taking up bodies as experimental sites for political action, they design an infrastructure (Yellow Dust DIY Sensing Infrastructure) whereby publics can experience air pollution in “embodied, collective and relational ways”, making invisible particulate matter (PM2.5) “sense/ible” (2019, p. 340). It is through these “molecular intimacies” (citing Chen 2012) that Calvillo and Garnett say new forms of engagement are produced: “constructing the problem collectively means the solution (action) can also be different” (citing Stengers 2000, p. 59, in 2019, p. 343). Here, then, I use arts-based workshops in a “cat-cradling” of bodies and things to map and intervene in how drug use, dependency, and recovery gets known and disrupted.

## Method: mapping chemical bodies

Bodies, unlike being and subjectivity, hone-in on the here and now, and circumvent claims on interiority (Agard-Jones 2013), allowing us to think about the human and nonhuman entities that make them up and put them into motion (Deleuze and Guattari 1987; Shapiro 2015; Povinelli 2016; Roy 2018). Bodies as assemblages of organic and inorganic matter are “a melding together of blood, bones, flesh, chemicals, chromosomes, cells, and spirit” that bare the traces of various forms of power as they are in/capacitated to act (Agard-Jones 2013, pp. 16–18). As such, I have found them to be a particularly useful tool and guide for registering and imagining new ways of relating to drugs. Extending my previous work on body mapping in the interview as a mode of “string-figuring” (Haraway 2016, please see Dennis, 2020, for more details), I take this method and metaphor further here as a way of expanding the number and kinds of participating bodies. For Isabelle Stengers, who is also an important thinker for this study, this methodological “cat-cradling” is vital for thinking differently; to be activated by someone or something is to avoid reproducing the same points, which means there is always an intervention (Savransky and Stengers^ 2018^, p. 131).

Over a series of four 3-h workshops in the summer of 2019 (and one additional 2-h follow-up session in 2021), seven participants were invited to describe their experiences of ‘living with’ and ‘without’ drugs (predominantly, heroin, crack cocaine and/or alcohol).^1^,^2^,^3^ These terms, ‘with’ and ‘without’, were purposely used in the workshop information and delivery to avoid the heavily narrativized and co-producing terms of addiction and recovery (Keane 2002, p. 158; Pienaar and Dilkes- Frayne 2017). The participants were all abstaining or trying to abstain from drug use and attended the same London (UK) drug service. Notably, three out of the seven participants also attended Narcotics or Alcoholics Anonymous fellowships which exist outside of formal treatment structures and are strictly abstinence driven: “the one requirement of membership is a desire to stop using” (UKNA 2021). I met all the participants while carrying out background ethnographic observations at the service in the weeks leading up to the workshops. With the service manager and keyworkers, I reached out to those who we thought would be interested in taking part and would offer diverse perspectives, taking into account their ethnicity, gender, age, stage of ‘recovery’, and the drugs they use/d. Except for one participant who was a photographer, none identified as having an arts background and no previous experience of attending arts-based workshops was required.

**Fig. 1:**
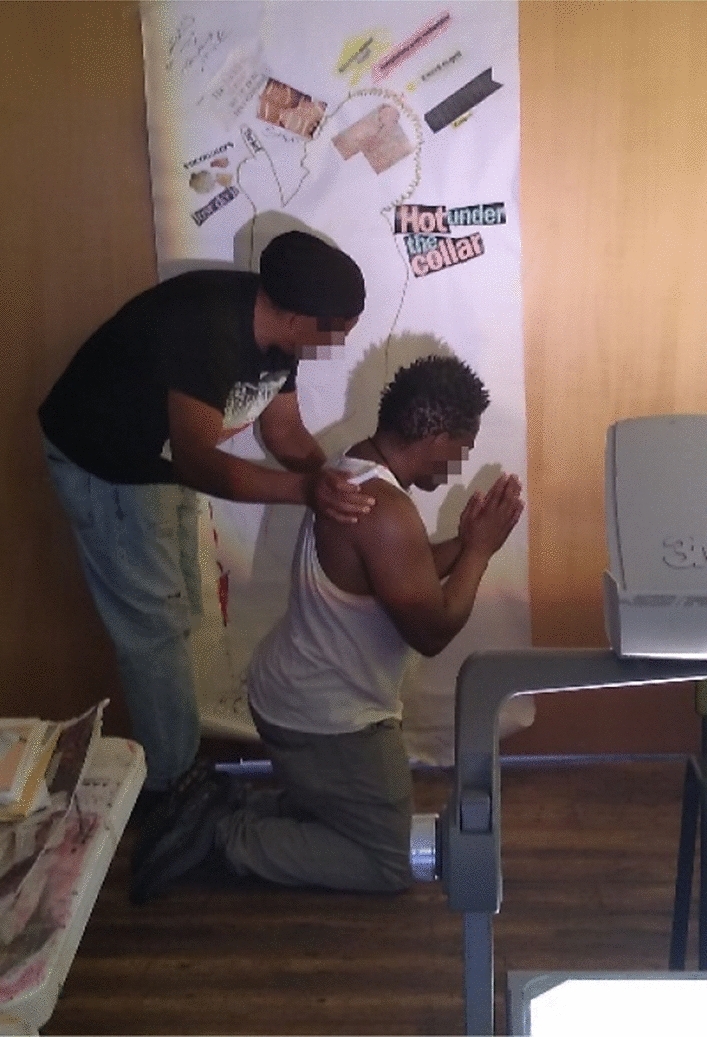
Participants, Jamil and Lennox, drawing the second outline for ‘living without drugs’. Jamil takes a kneeled, prayer position to mark the everyday practice of “handing over will” and paying attention to this vulnerability. We can just make out‘the map’ in Jamil’s representation of the topography of drug use, which gets stuck to the head of his body map (top centre of the photo).

In pairs, participants drew two outlines of their bodies using the shadow cast by an overhead projector (see Fig. 1), which offered flexibility in terms of the bodily pose they could make (rather than having to lie down on the floor, typical of traditional body mapping, e.g. see Soloman 2002). The first outline was used for describing living (feeling, acting, thinking) *with* drugs (e.g. how substances may have structured their days, hindered, or enabled moods, and changed relationships to people, time, and space), while the second was used for describing living *without* drugs (e.g. how they have adapted, and how their activities and relationships to people, time and space have changed). Participants were then invited to populate these outlines using various collaging materials which were brought in by the participants, an artist working on the project (Isla Millar) and myself. I was particularly interested in how the two bodies intersected, and five out of the seven participants chose to layer them in the same picture to highlight this interaction (e.g. see Fig. 1).

Inspired by method as a form of cat-cradling, the purpose of the research, in this exchange of bodies and things, was not only to register but enliven and enact chemical entanglements, getting to know them precisely by putting them into play. To give an example, one participant brought in a piece of ‘gauze’—an enmeshed piece of metal wire broken off from a scouring pad—to tell a story of repetition, of continually running out of this material which is vital for assembling the crack pipe. But rather than stopping there, as the gauze entered the room, it triggered a series of responses and stories. Alicia says: “When he brought the gauze in, I started sweating”. She had a visceral response as her body is reminded of smoking crack cocaine. Lennox, on the other hand, was prompted to talk about the skill, technique, and taste involved: “Don’t burn it the way you burn it. Everyone’s got their own special way”. Identifying with this sentiment, Jamil confirms: “The amount of time spent on scraping it and wiping it and prodding it and just, there’s a lot of work that goes into it, you know”. Linda, then, remarks: “what you talking about gauze for?”, which sets Jamil up for a joke: “it’s to wash your pots and pans after you’ve had a smoke”, and the group laughs. At the end of this back-and-forth, Jamil asserts: “see, there’s a lot of stories around a little bit of metal!”.

Mapping bodies as a collaging method in the workshops allowed for these bodies and things to interact and tell stories. Moreover, in a further act of relay beyond the workshops, I have been working with two artists who have been responding to the body maps through various making practices and developing them into an 3D installation for exhibiting to and bringing members of the public into this onto-epistemological exchange (more details to follow).

## “I am still a work in progress”: the art of living with(out) drugs

I’ve got a way to go, I am still a work in progress.

Jamil

Jamil, here, is summarising a conversation on the ongoing and unfinished nature of living a life without drugs where they have previously featured heavily, organising time and space. For Jamil, drugs were always there even when they weren’t there. He grew up in what he describes as a “drug area” and was getting repeatedly stopped and searched by police, labelled a drug user long before he started using them: “I’d be getting searched like how much times a day, before I even started with the drugs”. He sticks a street map of London to his body map (Fig. 1) and describes getting caught up in this “map” of use:

I would go ‘round this map, calling people, saying that I went to work, somebody stole my bag and it had this and that, borrow some money to get a cab so I could go up to my brother [and buy drugs] – all these stories I came up with […] Even after that [having consumed drugs, notably crack cocaine and heroin], I’m on the road, and on this map again.

Entwined in this topography of drug use (raising the funds, buying, and using substances), getting off and staying off “the map” involved making new connections and going to new places he describes as previously “out of bounds”.

For many participants, drugs had become an embedded part of their everyday and “normal”: “I just can’t deal with reality. I just couldn’t deal with being normal. I’ve always had a drug in me. I’ve never been clean even for one day” (Nick). Participants talked about how drugs enabled them to “close off”, wear a mask, “armour up” and “block out”: “I closed off to the world” (Jamil); “I’ve always worn a mask” (Lennox); “It did a lot of the blocking out, the reality that I just didn't want to know” (Alicia). Describing a magazine cutting she had stuck to her body map, Linda says: “There’s this armour guy [referring to an image of a medieval knight in jousting armour]. That was me, acting. As if I’m coping with life”. Similarly, Dave sticks a piece of metal foil (used for smoking heroin) to his body map for reflecting the viewer’s image back to them and asks: “what is your pain and what are you trying to hide away from?”.

Trying to live without drugs, where they had always been (as we have seen, beyond the drug as a physical, stable, or clearly bounded substance [like in Jamil’s example], but where it exists in these ecologies), left a vulnerability and porosity that required vigilance: “You have to be vigilant about this stuff, you can’t be complacent”. Lennox confirms: “I’m under no illusion that my illness is for life, and my addict is just waiting for the right moment, until it tells me something”. “Today, everyday I’m on my knees”, says Jamil, “handing over will”, paying attention to this vulnerability (see Fig. 1).

Describing himself as a work in progress—a term used by artists to describe incomplete and ongoing projects—Jamil is not only highlighting the fragility and endurance of this work, but also its artfulness. Here, I am thinking with Stengers (above) notion of paying attention as an art that is skilful and requires a ritual. Or as Otis put it: “You realise that it’s continuous, life is about learning”. For these reasons, the term “work in progress” resonated strongly with the group and they decided it should be the title of the exhibition of the workshop outputs. But this “work in progress” must not be confused with an individualised notion of recovery, but rather the participants point to its intersecting *collective*, *embodied* and *kinetic* (ritual-like) qualities.

Speaking on the collectivity of this ‘work’, Linda states (quoting from a magazine excerpt which she stuck to her body map): “I am where I am because of who *we* are”. Explaining the importance of other people (the “we”) in learning to live without alcohol, she states:

That means, I came to [the drug service] and *we*, collectively – if it wasn’t for [the drug service], I wouldn’t have got into recovery and gone on to the [Alcoholics Anonymous] Fellowship. So it’s a collective. Wherever *I* go, it’s always a *“we”*. Because I’m not doing this by myself. Without these lot, I wouldn’t be here.

With the help of this magazine cutting, in an act of cat-cradling, Linda points to the *collective* work of recovery. She explores the role of the drug service as not only a place to access formal support, but a place to meet people and collectively heal together. Linda and others talk about their peer mentorship and volunteering roles as a way of forming new structures and ways of thinking and being. Otis says: “having a bit of structure there helps because one of the things I’ve learnt is not to over-think stuff”. For Otis, attending the service is a way to redirect his thoughts by forming new temporalities and spatialities, or, to get off “the map”, in Jamil’s phrasing. Jamil speaks fondly of throwing himself into various voluntary positions working with other people affected by drugs and the prison system. For Jamil, working on himself in “keeping safe” is intrinsically about working with others and gaining energy from these relationships.

**Fig. 2:**
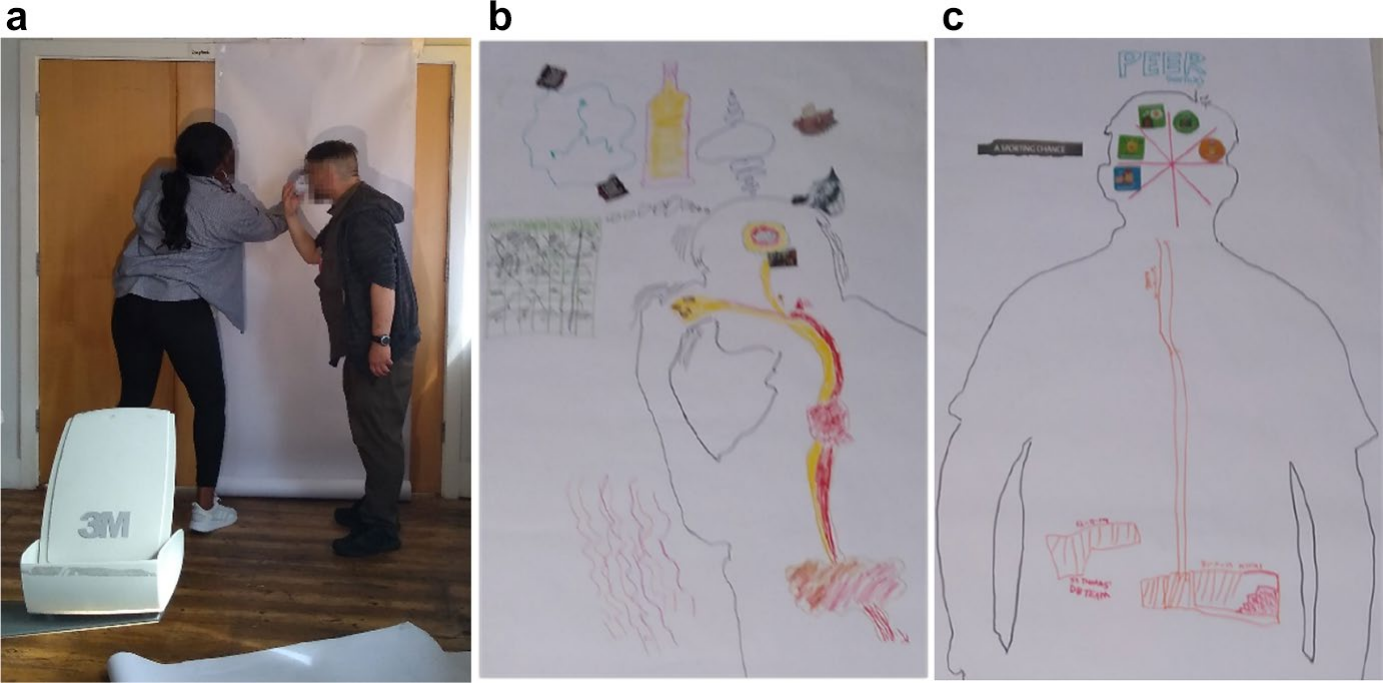
2a: Drawing an outline. 2b: “The wine (yellow) going down and the blood (red) coming up”, and a “chaotic” calendar (left). 2c: “Becoming a series of dates at the hospital” (liver and kidney drawn as hospital buildings), and a compartmentalized head.

These artful practices and forms of chemical kinship (Agard-Jones 2013) were also *embodied*. Otis, for example, describes a silent take-over in which alcohol had altered the functioning of his internal organs (see Fig. 2). As Balayannis and Garnett (2019) remind us: “Stories of chemical kin are not necessarily affirmative, kin after all can be both enabling and harmful” (no page). Otis recalls: “That’s the wine going everywhere and into my brain, and down into my various parts—*it was affecting me without me knowing*. My liver and various bits”. That is, until: “this is the wine going down and the blood going up, just before it all went pop” (Fig. 2):

My liver stopped working very well. My varices – the veins that go up in your neck – essentially, popped. And one morning I woke up at about five and filled the sink full of blood. I vomited up loads of blood, lots and lots of blood. I was losing blood at both ends.

Although this is an extreme example, it highlights the *porosity of bodies made with substances*, “*becoming with* and *orienting toward* molecular constituents” (Shapiro 2015, p. 374, original emphasis). Participants describe practices of reattuning to close off these leaky systems. For example, in Otis’s “living without” picture (Fig. 2c), he draws his liver and kidneys as hospital buildings, noting: “I’ve become a series of dates at the hospital”. Bodies as assemblages of organic and nonorganic matter and knowledges have to be worked at. He now has to strictly abstain from alcohol, engage in regular exercise, and attend frequent hospital appointments where clinicians closely monitor his liver for signs of growth.

In other examples of this embodied “work”, Nick says: “I never used to eat a lot but I’m eating healthy now, I eat like avocado and stuff… 5-a-day, doing yoga…”. He uses the body mapping to explain how he had neglected his body and eats “healthy” as a way of restoring energy and vitality back to his body that had become “weakened”. Linking these embodied practices to thought, in a literal translation of the slogan, Nick writes in bold letters onto his body map: “food for thought”.

Drawing on the body as a vital register, participants talk about moving and exercise—stressing the kinetics of this “work”—to externalize and expel certain chemical exposures and responses that leave them feeling tense. For example, multiple participants talked about the surges in adrenaline that came from having a “busy brain”. Where drugs such as heroin and alcohol had been used to suppress or mediate such chemical responses, these exposed bodies now sought new ways for rerouting this kinetic energy. Nick says: “I wanted to shut off that head. My brain, going round and round and round. And the only thing at the time was drugs. It wasn’t the right thing to do but…”. Now, suggesting little choice and a sense of urgency: “I’ve got to go for a walk, I’ll walk round and round the block like a mad man, like a horse. But once I’m out and going, I can calm my brain down”. Resonating, Jamil responds: “that’s why I run, to clear my head”. Linda opens herself up to new thoughts through these embodied practices: “Now, today, I meditate. I like to walk and meditate. Look at all the bright colours, nature… because today I can be in my own head and be comfortable”. Unsure how to negotiate these energies in the early days of trying to live without drugs, Lennox saw no other option but physical containment: “My escape route was to go to prison. *Educate myself in being bored*’.

Having looked at some of the ongoing and artful (embodied, collective, kinetic) work involved in paying attention to and rerouting chemically transformed bodies without drugs, I now turn to where this work can feel tiring and even threatening, intersecting with and feeding back into oppressive histories and political economies.

## Staying alert: “It’s hanging over you!”

Jamil: There’s a thing what they say about people who relapse, they say “they wouldn’t do this and wouldn’t do that” – and there’s this “and yet”. […] If you don’t deal with your ‘stuff’, that these things are yet to happen…

Linda: *They* say – we’re going to get into a debate – because it’s either going to be the “and yets” or the “agains”. It’s either a “yet” or we just do it “again”.

Jamil: But it ties to “*work in progress”*, innit. Because “yet again”, is still… […] “Wow, I didn’t really think, if I’m not more *vigilant*!” [I could become an addict]

Linda: (getting annoyed) Some people don’t have “and yets”, and some people don’t have “agains”, that’s why I’m being a bit of a stickler about it.

Fay (researcher): Are these terms that…

A few participants say together: Fellowship. ‘NA’ and ‘AA’

Linda: If you don’t follow this, *then*... If you don’t follow that…

Dave: …then you’ll relapse

Linda: You are *yet* to lose your house, your partner, your …

Alicia: That’s a threat, that is!

Linda: Well, exactly!

Alicia: Oh, come on, there’s no need for it. It’s hanging over you!

In this excerpt from one of the workshops, participants enter into a heated debate over whether it is okay to conceive of “the work” involved in living without drugs in this negative way, that is, to be performed out of a fear over *what is yet to come (again)* if one fails to pay attention and “deal with [their] stuff”. With regard to the exhibition, the participants debate whether this aspect of being a “work-in-progress” should be the basis for building connection with the public (grounded in the idea that anyone could become an addict). Linda and Alicia actively oppose this way of living in fear of a future that loops one into a seemingly inescapable past. Alicia imagines this relationship as a figure that “hangs over you”. To explore this sentiment further, I will first look at this oppressive imagery as a shared concern among the group, before linking it to participants’ personal narratives and histories, and then turning to an alternative practice of “blocking out”.

**Fig. 3:**
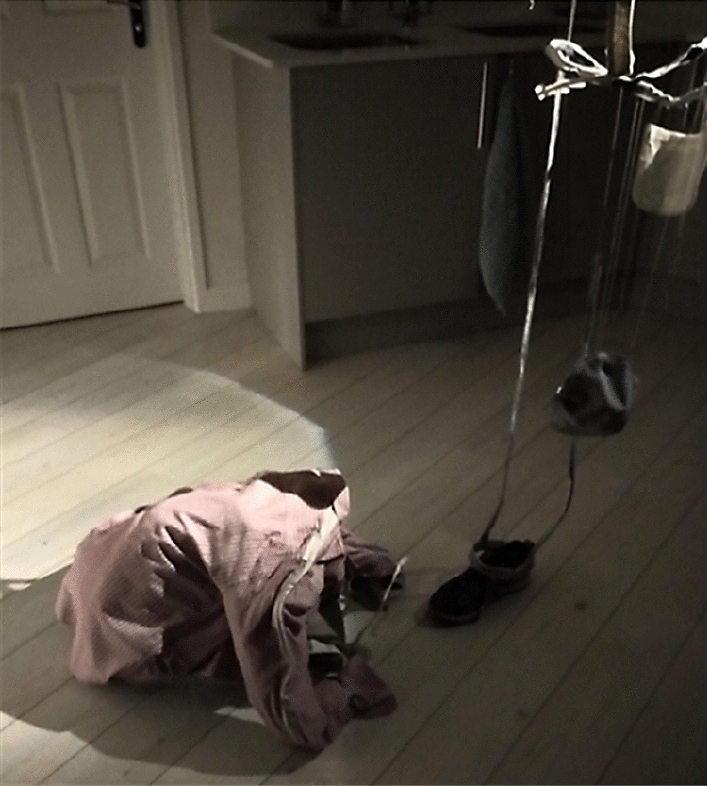
“It’s hanging over you”. A figure made while thinking through the data. [This is not a finished piece] Photograph taken by Isla Millar. ©Isla Millar & Penny Maltby.

The image of a figure that “hangs over you” is similarly evoked in Lennox’s (above) depiction of his ongoing relationship with drugs: “My addict is just waiting for the right moment, until it tells me something”. By personifying these attunements into a character that lurks, waiting for an opportune moment to surface, Lennox goes on to explain how he must be “on guard”. Likewise, Otis says: “I need to remind myself around my illness. Just try and *stay alert*, as best as I can”. Working with artists in generating data, these invisible forces get materialised in the making process (see Fig. 3). In engaging with the artwork, we can see and feel the precarity of these chemical relations that are already embedded in an ecology or what Murphy (2008, above) calls a “chemical regime of living” that makes such “work” exhausting and oppressive.

Gesturing towards the porosity of a life once lived with drugs and the “work” involved in living without them that can turn into something more sinister, Jamil says (also see Fig. 1):

the big part of my Fellowship is having faith and me *handing over my will* every morning (in prayer), hand over my will to a god of my understanding, not […] some hippy looking Jesus, looking like Michelangelo’s interpretation of him […] because these are the images that were rolled out to suit a racist and chauvinistic perspective, you know, but that’s the deeper side.

Noting this “deeper side” of where these chemical kinships can become colonised by what Jamil calls a racist and chauvinist perspective, he draws our attention to the politics of these recovery practices that can quickly rigidify or turn “molar”. If we are not careful, this artful work in generating and maintaining fulfilling and creative lives without drugs can become co-opted. It was vital, therefore, to Jamil that there was manoeuvrability in how he adopted treatment principles, in steering his recovery and taking account of his particular history: “If I don’t know my history, I’d be condemning myself”. He is referring, I believe, to the biblical texts and racist context of 1930s United States where the 12-Step Fellowship originates. In this, casting threads across time and space, Jamil is making connections between and noting the ongoing entanglement of these colonial and racist projects and modern-day recovery and treatment.

Next, I want to look at some of the histories that participants understand as being connected to their drug use but can get obscured by recovery practices that focus too much on individual autonomy. Following a description of adverse life experiences in which he had been repeatedly subjected to the racist police practice of ‘stop and search’ that labelled him a drug user before he had even started using them, suffered a close bereavement and witnessed domestic violence, Jamil says, fearing the group is losing concentration: “All of this stuff, it does connect. There is a connection!”. Nick states: “I got very angry with the world and that was why I turned to the drugs”. The *negative* forces that introduce and retain people in drug consumption and define them by this practice are well known to be unequally distributed, determined by historical and political economic forms of violence. Participants accounted for their drug use in terms of feeling let down and disconnected from/by society (and family): “I feel I was let down” (Dave); “I was in the care system as well, so I never felt connected. It was me against the whole world” (Linda). Trying to reconcile his experience of harsh parenting, Lennox imagines “what it was like for them, my parents, coming over here [from the Caribbean]. Not that I blame [them]…”. Speaking about having to move across London to live with a father he barely knew, Jamil says “Nobody would have me”. And, for Dave, reflecting on this unmet sense of connection: “What I need is love, because as a child I didn’t get any love”.

Within this context or ecology, we can start to really appreciate the conviction and importance of Alicia’s interjection in the discussion above. Her statement can be read as a refusal to pay attention to this vulnerability in the way Jamil (via the Fellowships) as well as Lennox and Otis are suggesting. She does not want to live in fear of what is yet or again to come, that is, the losses—“your partner, your house”—and abject behaviours that get associated with addiction. In this sense, she is highlighting how being a work-in-progress can get felt as threatening and fails to appreciate these unequal histories that mean some people may be more likely to use drugs but, more importantly, be defined and negatively affected by them. Therefore, in refusing to pay attention at the individual level to these chemical ecologies that have defined and confined her for too long—to the addict figure that lurks or hangs over her—Alicia is refusing this identity and politics.

Aware of the potential risks of this embodied reflection (where she continues to be chemically attuned) but also the politics in which it is enfolded, Alicia prefers *not* to pay attention.

But you’ve got to remember, Fay, you know, after six, it’s evening, isn’t it!? And you know, for me, it’s going to bring up feelings, and I’m going to go home, on my own, with that in my mind. So, I’m not going to go into depth, not today.

In this refusal to take part in some aspects of the workshop, Alicia chooses not to remember, at least not on demand and without sufficient time to then un-remember. For Alicia, this kind of paying attention plays into a violent history of paying attention to parents that abused and neglected her, teachers that belittled her and made her feel inadequate, partners that were cruel and beat her, clients that humiliated her and treated her like an object, police officers that manhandled her, social workers who she opened up to for support but took her children, and many other forms of oppression she has had to endure in her life. Alicia talks to me in private about horrific acts of abuse and betrayal. In a further enactment of this refusal to pay attention to these chemical ecologies, she refuses to continue seeing those around her who continue to consume drugs: “I ain't putting up with his shit. I ain't doing it”; “I block it out”; “That would bring you down like that, mate. I can't do it”.

To draw on Elizabeth Robert’s point again, where “life is already deeply entangled with chronic economic and political instability”, we need more critical accounts of entanglement (2011: 616). Alicia doesn’t want to be stuck in this constant work of never feeling complete and always living in fear of unravelling—under the shadow of what she is “yet” or “again” to do. In other words, being a work-in-progress is not good enough. She does not want to pay attention anymore. As such, she chooses not to revisit these memories or be confronted by those in her family who continue to consume drugs. “Blocking out”, rather than paying attention, is a mechanism that has helped her, and recognises a different mode of survival and living in unequally entangled, drugged worlds.

## Discussion: not paying attention in the face of toxic entanglement

In her essay, *In Catastrophic Times: Resisting the Coming Barbarism*, Isabelle Stengers (2015) lays out an intervention for surviving our current late industrial epoch defined by demands for economic growth and progress. Key to this is re-learning the art of paying attention and joy of being “in the middle”. Paying attention “creates an obligation to imagine, to check, to envisage, consequences that bring into play connections between what we are in the habit of keeping separate” (2015, p. 62). This can be seen extended in accounts such as Anna Tsing’s (2015) “arts of noticing”, where she captures anti-progress world-making. Where addiction, as a biomedical category (illness/disease) closes-down and shuts off ways of being with drugs as a form of neurological defect, chemosociality offers an alternative account of living with(out) drugs that fosters intrigue rather than pathology and an awareness towards how we are folded into our chemical environments. Revisiting Deleuze and Guattari (1987)’s notion of the molecular, chemicals help to free up our ‘molarised’ models of drug use such as addiction. Indeed, this might be the very kind of “alternative and non-pathological form of attachment” that Helen Keane recently imagines in “a future of addiction that is not destructive of individual and collective wellbeing” (2021, p. 3). Therefore, I return now to the pressing question laid out at the beginning: *In what ways might chemicals erode how we think about addiction?* And furthermore: *How may they offer ways of living in the face of toxic chemical entanglement?*

By putting bodies into play in workshop settings, participants reveal collective, embodied, and kinetic practices of paying attention to chemically altered bodies where chemicals have been used to “block out” (Alicia), “wear masks” (Lennox) and “armour up” (Linda) against the intersecting violences of prohibition, poverty, racism, sexism and colonialism, seen, for example, in racist policing, inadequate State services, neglectful/abusive parenting, hostile immigration environments, and domestic violence. Returning to Roberts’s ethnography of “what gets inside” in Mexico City, she documents how drugs can work to secure “stability through toxic boundaries” (2011, p. 614). Living without drugs then, participants identify an incompleteness where there is an ongoing struggle to stay aware of the places, emotions, feelings, times-of-day, and thoughts where these chemical attunements lurk (characterised as “the addict”). While this process is artful like in Stengers definition of paying attention, there is also a darker side. Where some participants embrace themselves as works-in-progress and always “on guard”, others are more cautious and refuse the threat-like form this “work” can take. With this, the work can play into and cover over oppressive political economies and histories that mean some people, particularly those marginalised by race, gender, and class inequality, are more likely to be defined and confined by drugs, even if they do not use them, as Jamil highlight). Looking for more ethical ways out of ‘molar’ addiction thinking and treatment that can recognise these inequities, I conversely return to participants’ “blocking” practices.

Rather than trying to overcome or confront “blocking” practices that are popular in narratives of recovery and rehabilitation, I think of them  here as an alternative mode of surviving and living in unequally entangled worlds. By not paying attention to “the addict”, people who use/d drugs are refusing those processes that individualise addiction and the forms of oppression it intersects with and perpetuates. There are similarities here with recent activist practices of “not doing” as a refusal to participate in capitalist temporalities that demand a constant doing:

[We embrace] unproductivity as an activist practice and the ways in which caring, resting, suspending, pausing and breaking can be re/claimed as political acts by and for everyone, particularly those marginalized by the racial and gender inequalities of neo-liberal capitalism (‘Art of not doing’ conference, 2019).

In this sense, “blocking out” goes even further and can be reimagined as an affirmative practice for living in unequally drugged worlds.

In the heated discussion over the terms in which to engage people in “the work” involved in living without drugs, both in the Fellowships and in the exhibition of the participants’ body maps, Alicia and Linda explicitly reject the idea of building care based on fear: the abject and harmful things that one is yet to do (still or again). Regarding the exhibition, building a connection grounded in fear—that anybody could become an addict—turns care and our questioning inward into a concern for oneself, asking: could I become an addict? In art-science projects, this has become a popular ‘hook’ and mechanism for engaging publics on issues of drug dependency. For example, in artworks like “Feed me” by Rachel Maclean (2015) and the recent exhibition it was staged in, “Hooked: When Want Becomes Need” (Science Gallery London^ 2018^), they rely on a fear of addiction based on a fear of a malfunctioning capitalist system or consumer compulsion. In turning the terms of/for care inward, such interventions point to where Eva Giraud suggests an expression of entanglement may actually curtail responsibility: “Be careful that an emphasis on complexity and uncertainty doesn’t foreclose rather than open-up responsibility in certain contexts” (2019, p. 96). With this, I am reminded of the difference between concern and care as posed by Maria Puig de la Bellacasa, where.

the second adds a strong sense of attachment and commitment to something. Moreover, the quality of care is more easily turned into a verb: *to care*. One can make oneself concerned, but ‘to care’ more strongly directs us to a notion of material doing (2011, p. 89, original emphasis).

Building care rather than concern, then, the planned exhibition intends to affect, touch, and bring visitors into these precarious worlds where they will move within and between 3D figures of fragmented, tenuously held together bodies (like that seen in Fig. 3). As one of the artists, Isla Millar, put it, this is not to provoke sympathy but empathy. Empathy, originating from the German, ‘Einfühlung’.

literally means “feeling into” and refers to an act of projecting oneself into another body or environment […] which is aimed at understanding what it would be like to be *living another body* or another environment. (Ganczarek et al. 2018, emphasis added)

Where sympathy relies on feeling sorry for somebody else, creating a distancing effect between the self and other, empathy, on the other hand, relies on getting closer to and projecting oneself into the body of the other, to get a feel for and become-with them. Moving and feeling within these 3D bodies, therefore, may help to push our understanding of different ways of living with drugs’ ongoing affects where “blocking” may not only be a legitimate way of living without drugs or doing recovery after addiction, where paying attention has become unequally exhausting, but an affirmative rather than defensive practice.

Taking up the collaging materials in a playful approach to knowledge production through making bodily figures and inviting publics to engage with them in the exhibition, we hope to transform these chemical kin and ecologies into matters of care (Puig la Bellacasa 2011). With this, we hope to add care to a concern for people who use/d drugs: “matters of care is a way of relating to them, of inevitably becoming affected by them, and of modifying their potential to affect others” (Puig la Bellacasa 2011, p. 99). Rather than feeling ‘concerned for’ people who use/d drugs, visitors are encouraged *to care* as an embodied act of being affected and affecting. Where, as one scholar pointed out recently, there is a distinct lack of care towards people who use drugs—asking in one national UK newspaper, “be honest, do you actually care about the record number of drug-related deaths?” (Hamilton 2019: no page)—this may be exactly the kind of restorative work needed to open-up discussions around how we treat and respond to people who use/d drugs. By putting publics *in touch* with these lives in an act of cat-cradling—of bodies, things, and affects—we can get a *feel* for this precarity in making them ‘sensi/ible’ (Calvillo and Garnett 2019). In this, we become part of and make possible alternative ways of living with/out drugs that do not have to rely so heavily on fear (of the addict). In a treatment system that is often time-limited and considered successful as soon as the person is “drug free”, we hope the exhibition, in some small way, helps to bring about longer-term support and diversify what success and recovery means and looks like for those involved.

## Conclusion

At the start of this article, I quoted a participant who set in motion the study’s aim to find where drugs and bodies conjoin in infinitely complex and seemingly senseless ways, to identify more ethical ways forward. In her book, *What Comes After Entanglement?*, Eva Giraud argues for the importance of care in “reintroducing (perhaps unfashionable) critical questions about how to respond to irreducibly entangled worlds” (Giraud 2019, p. 104). With this in mind, participants here are not only finding better ways of living with chemicals as they continue to make themselves known, but draw our attention to the problem of this entanglement as it presses down on bodies unequally. As “works-in-progress”, participants describe the precarious and artful work of living without drugs as a mode of living with their ongoing affects, paying attention to where these chemical attunements lurk. But while these skills of paying attention are artful in Stengers’ sense, ritually attuning them in collective, embodied, and kinetic ways to what may lurk and be allowed to flourish as a result, they can become exhausting and easily co-opted by oppressive, ‘molarising’ forces (“it’s hanging over you!”) that have defined people who use/d drugs for too long and disguise social and political failings as individual ones.

Therefore, my argument is twofold. While understanding drug-recovery practices as sociomaterial processes of paying attention to chemically-altered bodies (as works-in-progress) goes some way to de-pathologising and ‘molecularising’ addiction: to “traverse, create a path, destabilise, enable energy seepage within and through these molar unities” (Grosz 1993, quoted above). I also hope to have made space for *not* paying attention as an alternative way of coping and living in unequally entangled worlds. Where scholars in the ‘chemical turn’ ask us to explore these situated examples of living in, routing paths through and “finding the joy” in infinitely complex and troubling worlds, here, we see how people who have/had drug dependencies also turn to practices of distraction and avoidance—“blocking out” and “educating [themselves] in being bored”. In this sense, this adds to critical turns and alternative ways of living in chemically transformed worlds defined by a drive for progress (e.g. “alterlife”, Murphy 2017). It is here that not paying attention may be one such mode of living “after progress” (Savransky and Lundy 2022): refusing to be a “work in *progress*”. Furthermore, in a world where we cannot disentangle drugs and drug dependencies from their political economies and histories, it is my proposal that a chemical reading of peoples’ relationships with drugs might provide a more capacious, politically engaged, and caring way forward for living with and responding to drugs *after addiction.*

## Footnotes

1 I use the term ‘drug’ to include alcohol and recognise alcohol as a type of drug.

2 Informed consent was taken at the beginning of each session and all participants consented to having photographs taken and shared for research purposes.

3 This study was granted ethical approval from the Research Ethics and Integrity Sub-Committee at Goldsmiths, University of London (Ref: 1366/1), and the National Health Service London (Harrow) Research Ethics Committee (Ref:18/LO/1333).
